# HLA-B and TIMP1 as hub genes of the ventricular remodeling caused by hypertension

**DOI:** 10.18632/aging.205816

**Published:** 2024-05-09

**Authors:** Gai-Feng Hu, Ling-Bing Meng, Jianyi Li, Jiapei Xu, Hong-Xuan Xu, De-Ping Liu

**Affiliations:** 1 Department of Cardiology, Beijing Anzhen Hospital, Capital Medical University, National Clinical Research Center for Cardiovascular Diseases, Chaoyang 100029, Beijing, China; 2 Department of Cardiology, Beijing Tsinghua Changgung Hospital, School of Clinical Medicine, Tsinghua University, Changping 102218, Beijing, China; 3 Department of Cardiology, Beijing Hospital, National Center of Gerontology, National Center of Gerontology, Institute of Geriatric Medicine, Chinese Academy of Medical Sciences, Dong Dan 100730, Beijing, China; 4 Graduate School of Peking Union Medical College and Chinese Academy of Medical Sciences, Dongcheng 100730, Beijing, China

**Keywords:** hypertension, myocardial fibrosis, ventricular remodeling, significant module, HLA-B

## Abstract

Rationale: Myocardial fibrosis is an important pathological change that occurs during ventricular remodeling in patients with hypertension and is an important pathophysiological basis of cardiovascular disease. However, the molecular mechanism underlying this ventricular remodeling is unclear.

Methods: Bioinformatics analysis identified HLA-B and TIMP1 as hub genes in the process of myocardial fibrosis. Expression and correlation analyses of significant hub genes with ventricular remodeling were performed. Weighted gene co-expression network analysis (WGCNA) was performed to verify the role of HLA-B. ceRNA network was constructed to identify the candidate molecule drugs. Receiver operating characteristic (ROC) curves were analyzed.

Results: RT-qPCR was performed to verify the roles of HLA-B and TIMP1 in seven control individuals with hypertension and seven patients with hypertension and ventricular remodeling. The WGCNA showed that HLA-B was in the brown module and the correlation coefficient between HLA-B and ventricular remodeling was 0.67. Based on univariate logistic proportional regression analysis, HLA-B influences ventricular remodeling (P<0.05). RT-qPCR showed that the relative expression levels of HLA-B and TIMP1 were significantly higher in HLVR samples compared with their expression in the control group.

Conclusions: HLA-B and TIMP1 might provide novel research targets for the diagnosis and treatment of HLVR.

## INTRODUCTION

As a clinical syndrome characterized by high blood pressure in the systemic circulation arteries, hypertension can be accompanied by functional or organic damage of the heart, brain, kidney, and other organs [1]. Hypertension is the most common cardiovascular disease in the world and its prevalence is increasing. Hypertension is also the primary risk factor for cardiovascular and cerebrovascular diseases. Hypertension and its complications are ranked first in the causes of death globally, which not only seriously affects patients’ life expectancy and quality of life but also causes huge social harm and economic burden. Long-term hypertension can change the morphology and substance of the heart that can then lead to ventricular remodeling, which is an adaptive response of the body that can include a secondary pathophysiological process caused by lesion repair and overall compensation of the ventricle [2].

Myocardial fibrosis is an important pathological change that occurs in ventricular remodeling in patients with hypertension and is an important pathophysiological basis for cardiovascular disease [3]. Myocardial fibrosis in hypertensive patients is accompanied by inflammatory cell infiltration around the coronary arteries and myocardial interstitial tissue, while the chemokine-mediated immune and inflammatory responses play an important regulatory role in myocardial fiber remodeling, indicating that the chemokine-mediated inflammatory response is one of the important inducers of adverse cardiovascular events [3, 4]. Some studies have shown that the main pathological mechanisms of myocardial fibrosis in hypertension are inherited membrane ion transport defects, abnormal sodium metabolism, and dysfunction of renal sodium excretion, which lead to immune responses, oxidative stress, and inflammatory responses [5]. Systolic hypertension is more likely to induce oxidative stress injury, leading to inflammatory cell infiltration followed by an increase in cytokine secretion [6].

Ventricular remodeling caused by hypertension is a multi-gene hereditary disease, which is the result of a combination of hereditary genetic factors and environmental factors; however, its pathogenesis has not yet been fully clarified [3, 7]. With the development of bioinformatics technology, we have entered the era of genetic testing. Bioinformatics is a discipline encompassing the collection, processing, storage, dissemination, analysis, and interpretation of biological information. It is also a new discipline that combines the life sciences and computer science. Microarray analysis can simultaneously capture information relating to the expression of tens of thousands of genes and explore genomic changes related to the initiation and development of disease [8, 9]. Research into the genes involved in ventricular remodeling caused by hypertension is of great significance for understanding the mechanism of pathogenesis and the development of prevention strategies and will also provide a theoretical basis for molecular genetics for the precise treatment of ventricular remodeling caused by hypertension.

Therefore, this research aimed to utilize bioinformatics technology to screen differentially expressed genes (DEGs) between hypertensive patients with normal left ventricular size and hypertensive patients with human left ventricular remodeling (HLVR), using samples of white blood cells extracted from patients. The enrichment analysis of DEGs was performed using Gene Ontology (GO) and Kyoto Encyclopedia of Genes and Genomes (KEGG) analysis. A protein–protein interaction (PPI) network was also constructed. Subsequently, significant modules in the PPI network and significant hub genes were identified. Finally, the role of significant hub genes in ventricular remodeling caused by hypertension was analyzed.

## MATERIALS AND METHODS

### Obtaining public data

A dataset containing the RNA profiles of hypertensive patients with left ventricular remodeling (GSE74144) was downloaded from the Gene Expression Omnibus (GEO) database, and annotated using the platform GPL13497 (Agilent-026652 Whole Human Genome Microarray 4x44K v2). The GSE74144 dataset comprised 14 hypertensive patients with normal left ventricular size (Control, n=14) and 14 hypertensive patients with left ventricular remodeling (HLVR, n=14).

### Repeatability test of the data

Principal component analysis (PCA) can be used to divide mutually orthogonal and uncorrelated components. Therefore, the repeatability of the data was verified using PCA.

### Screening of DEGs

GEO2R is a system for the online analysis of data in the GEO database. DEGs between white blood cells in the control and the HLVR groups were screened using GEO2R. A P-value ≤ 0.05 and a fold change ≥ 1.5 or ≤ -1.5 were defined as the cut-off criteria. The DEGs were displayed as volcano maps that were drawn using a volcano plotting tool (https://shengxin.ren), based on the R language.

### Construction of the PPI network

STRING (Search Tool for the Retrieval of Interacting Genes, http://string-db.org), an online database, can be used to predict the protein–protein interaction (PPI) network of DEGs. Cytoscape (version 2.8), an open-source visualization software tool, was used to visualize this PPI network.

### Functional annotation of DEGs

Gene Ontology (GO) includes cellular components (CC), biological processes (BP), and molecular functions (MF). The KEGG database (Release 91.0, July 1, 2019, https://www.kegg.jp/kegg/) is an open database resource that allows the utilities and high-level functions of biological systems to be easily understood. The Database for Annotation, Visualization and Integrated Discovery (DAVID, v.6.8, https://david.ncifcrf.gov/) provides a well-rounded set of annotation tools for function and pathway enrichment analysis. The Biological Networks Gene Ontology tool (BiNGO) (version 3.0.3) was used to analyze and visualize the cellular components and molecular functions of the DEGs.

### Identification of significant modules and significant hub genes

The Cytoscape plug-in, Molecular Complex Detection (MCODE) (version 1.5.1), was used to identify the most important module in the network map. The criteria used for the MCODE analysis were a degree cut-off=2, MCODE scores >5, maximum depth=100, node score cut-off=0.2, and k-score=2. The hub genes in the PPI network were explored using cytoHubba with four arithmetics (MCC, MNC, EPC, and Degree). The common hub genes were obtained using a Venn diagram and delineated using the FunRich software (http://www.funrich.org/); they were then defined as significant hub genes.

### Expression and correlation analyses of significant hub genes

Heatmaps of the expression levels of common hub genes were visualized using the R language. Pearson’s correlation test was performed to complete the correlation analysis among the common hub genes. The construction of the heatmaps, which can display the correlations among all common hub genes, was completed using the R language.

### Identification of significant hub genes associated with ventricular remodeling

The Comparative Toxicogenomics Database (CTD, http://ctdbase.org/) was used to identify integrated chemical–disease, chemical–gene, and gene–disease interactions to predict novel associations and generate expanded networks. The relationships between gene products and ventricular remodeling were then analyzed using the CTD.

### Verification of HLA-B expression

The GSE74144 dataset was normalized using Perl. The R package “limma” was employed to calculate whether there was a difference in the expression of HLA-B.

### WGCNA

The WGCNA of all genes was performed using the R package “WGCNA” (https://cran.r-project.org/web/packages/WGCNA/index.html). A co-expression network for all genes was constructed, and the algorithm filtered 25% of the genes with the largest variation for further analysis. Then, 14 ventricular remodeling samples and 14 control samples were used for WGCNA. An adjacency matrix was created using the samples, and it was then transformed into a topological overlap matrix (TOM). Genes were divided into different gene modules using TOM-based difference measurement. A minimal gene module > 250 and a threshold to merge similar modules=0.1 were used to search for modules that play an important role in ventricular remodeling. In addition, WGCNA can be used to predict gene–gene interaction networks in the same module.

### Functional annotation of DEGs via Metascape and gene set enrichment analysis (GSEA)

The Metascape (http://metascape.org/gp/index.html) database contains a comprehensive gene-list annotation and analysis resource. In this study, GO and KEGG analyses of brown module genes were performed to explore the potential functions of HLA-B. Gene set enrichment analysis (GSEA, http://software.broadinstitute.org/gsea/index.jsp) is a computational method that can execute GO and KEGG analyses with a given gene list. In this study, all samples were divided into two groups based on the expression of HLA-B. We then performed functional enrichment to explore the potential mechanisms of HLA-B.

### Competitive endogenous RNA (ceRNA) for HLA-B prediction

starBase (www.starbase.sysu.edu.cn) is an online database that can be used to systematically identify RNA–RNA and protein–RNA interaction networks. In our study, starBase was used to screen for ceRNA networks that regulate HLA-B.

### Identification of candidate molecule drugs that target hub genes

The Connectivity Map (CMap, https://www.broadinstitute.org/connectivity-map-cmap) database can connect genes and genomic information with human diseases and drugs used to treat them. In this study, molecule drugs that could regulate HLA-B were searched for in the CMap database.

### The recruited patients and clinical data collection

A total of 14 patients were recruited and their serum samples were collected for RT-qPCR assay, comprising 7 control individuals with hypertension and 7 patients with hypertension and ventricular remodeling, and the baseline clinical characteristics of the two groups were collected from their medical records, which included haemodynamic parameters and co-medications of patients shown in Supplementary Table 3. Myocardial fibrosis detected by late gadolinium enhancement cardiovascular magnetic resonance (CMR-LGE) in hypertension patients by two cardiac radiologists was defined as ventricular remodeling group [10]. The hypertension patients without myocardial fibrosis by LGE-CMR were assigned to the control group without ventricular remodeling [3, 11]. The research conformed to the Declaration of Helsinki and was authorized by the Human Ethics and Research Ethics Committees of Beijing Hospital. Written informed consent was obtained from all participants.

### RT-qPCR assay

Total RNA was extracted from patient samples using the RNAiso Plus (Trizol) kit (Thermo Fisher Scientific, MA, USA) and reverse transcribed to cDNA. RT-qPCR was performed using a Light Cycler® 4800 System with specific primers for the hub genes. Supplementary Table 1 shows the primer sequences used in the experiments. The RQ values (2^−ΔΔCt^, where Ct is the threshold cycle) of each sample were calculated and presented as fold changes in gene expression relative to the control group.

### Statistical analysis

The data were expressed as a percentage of the total and mean ± SD. The statistical analyses in this study were performed using Perl, R software (version 3.5.3), and SPSS 20.0. The effect of HLA-B on ventricular remodeling based on univariate logistic proportional regression analysis was investigated using SPSS, version 24.0 (IBM Corp., NY, USA). A receiver operating characteristic (ROC) curve was plotted using MedCalc software, to measure the performance of HLA-B. Fisher’s exact test was used for categorical variables. For continuous variables, independent samples t-test (Student’s t-test) was used, and when the equal variances were not assumed, Brown-Forsythe was performed. A p-value < 0.05 was considered statistically significant.

### Availability of data and materials

The data used to support the findings of this study are available from the corresponding author upon request.

## RESULTS

### Data validation

**Figure 1:**
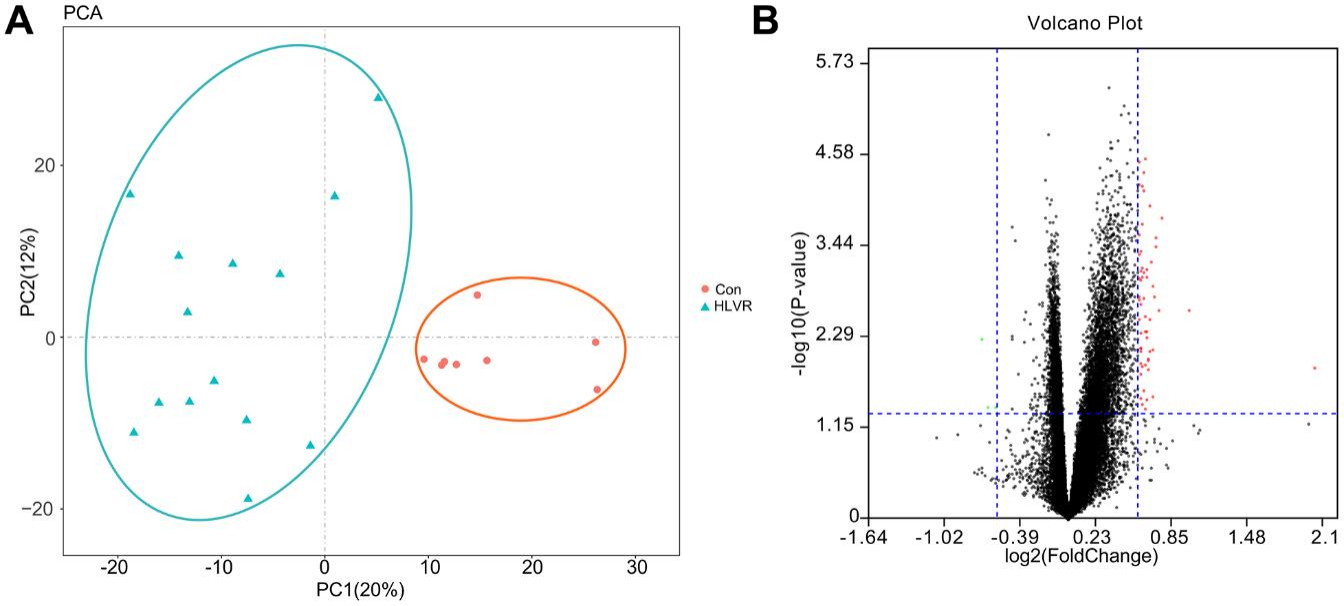
**The repeatability test for data and screening of DEGs.** (**A**) Principal component analysis. (**B**) A volcano map showing DEGs.

The GSE74144 dataset was obtained from the GEO database. After performing PCA, the repeatability of data in the GSE74144 dataset was shown to be acceptable. The distances between samples in both the control group and the HLVR group were close in the dimension of PC1 (Figure 1A).

### Identification of DEGs and the PPI network

**Figure 2:**
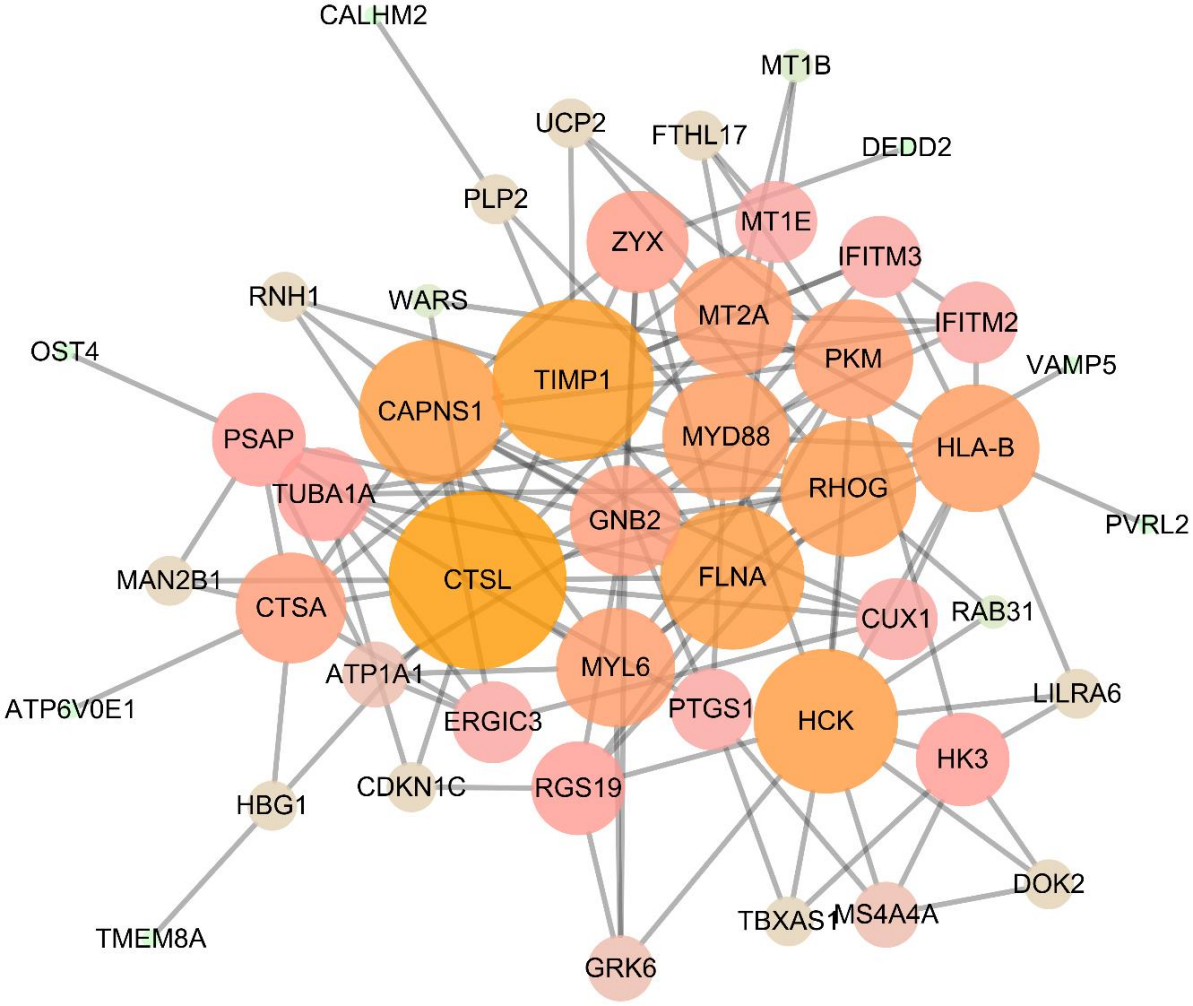
PPI network, including 128 edges and 47 nodes.

Through the GEO2R analysis, the DEGs between control and HLVR white blood cell samples in the GSE74144 dataset were shown in volcano plots (Figure 1B). The PPI network consisted of 128 edges and 47 nodes (Figure 2).

### Enrichment analysis of DEGs using DAVID

**Figure 3:**
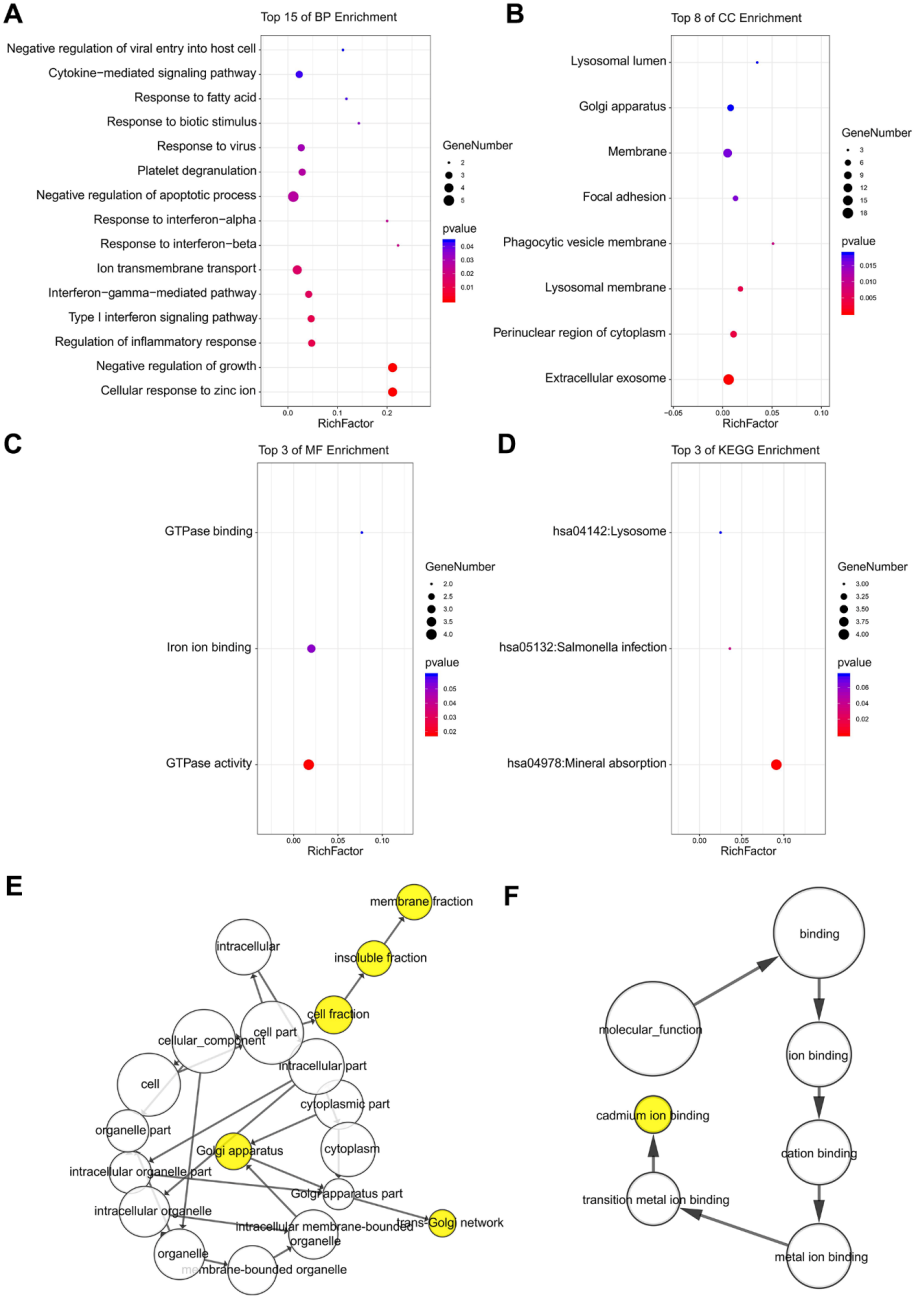
**The enrichment analyses of DEGs using DAVID and BiNGO.** Detailed information relating to changes in the (**A**) BP, (**B**) CC, (**C**) MF, and (**D**) KEGG analysis of DEGs via DAVID. Detailed information relating to changes in the (**E**) CC and (**F**) MF analysis of DEGs via BiNGO.

Following an analysis using DAVID, the results of our GO analysis showed that variations in DEGs linked with BP were mainly enriched in negative regulation of growth, regulation of inflammatory response, interferon-gamma-mediated pathway, response to interferon-alpha, and negative regulation of apoptotic processes (Figure 3A). Variations in DEGs linked with CC were significantly enriched in extracellular exosome, perinuclear region of cytoplasm, lysosomal membrane, phagocytic vesicle membrane, focal adhesion, and membrane (Figure 3B). With regard to MF, DEGs were significantly enriched in GTPase activity, iron ion binding, and GTPase binding (Figure 3C). Analysis of KEGG pathways indicated that the top canonical pathways associated with the DEGs were mineral absorption, Salmonella infection, and lysosome (Figure 3D).

### Further analysis for CC and MF using BiNGO

The CC and MF analysis using BiNGO for the hub genes is presented, and the results verified the results of the GO analysis (Figure 3E, 3F). The results of the BiNGO analysis showed that variations in CC were also mainly enriched in membrane fraction, insoluble fraction, cell fraction, and Golgi apparatus (Figure 3E). Changes in MF were also enriched in cadmium ion binding, transition metal ion binding, metal ion binding, cation binding, and ion binding (Figure 3F).

### Significant modules

**Figure 4:**
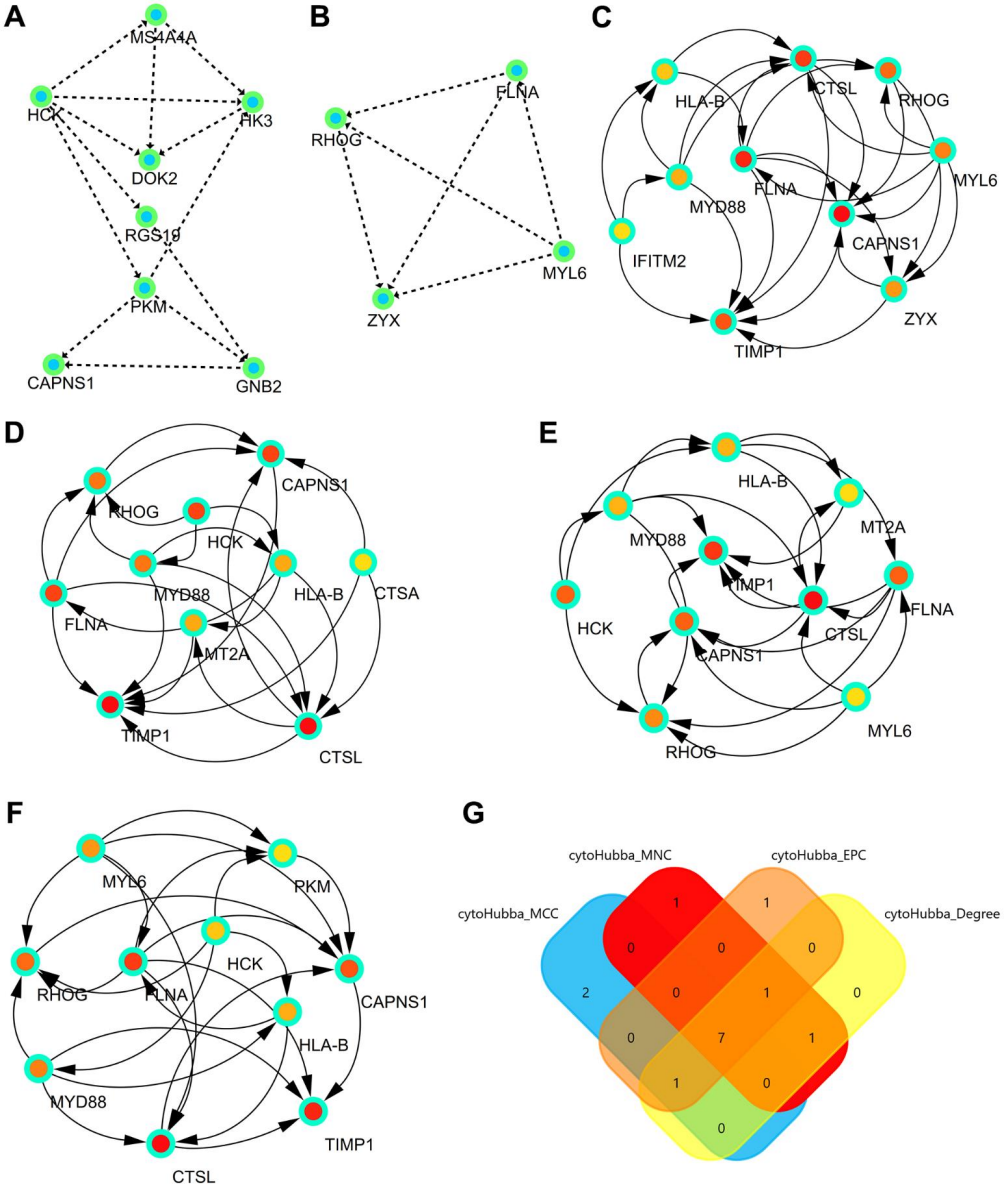
**Significant modules and hub genes identified from the PPI network.** (**A**) The first module consists of MS4A4A, HCK, HK3, DOK2, RGS19, PKM, CAPNS1, and GNB2. (**B**) The second module consists of FLNA, RHOG, MYL6, and ZYX. (**C**) The first network of hub genes identified by MCC. (**D**) The second network of hub genes identified by MNC. (**E**) The third network of hub genes identified by Degree. (**F**) The fourth network of hub genes identified by EPC. (**G**) The Venn diagram comprises the common hub genes (HLA-B, CTSL, RHOG, FLNA, MYD88, CAPNS1, and TIMP1).

The three most significant modules were obtained from the PPI network using Cityscape. The first module consisted of MS4A4A, HCK, HK3, DOK2, RGS19, PKM, CAPNS1, and GNB2 (Figure 4A). The second module consisted of FLNA, RHOG, MYL6, and ZYX (Figure 4B).

### Hub genes identified from the PPI network

Hub genes were identified by four algorithms, presented in Figure 4C–4F. Finally, the significant hub genes (HLA-B, CTSL, RHOG, FLNA, MYD88, CAPNS1, and TIMP1) were identified using a Venn diagram (Figure 4G).

### Analysis of significant hub genes

**Figure 5:**
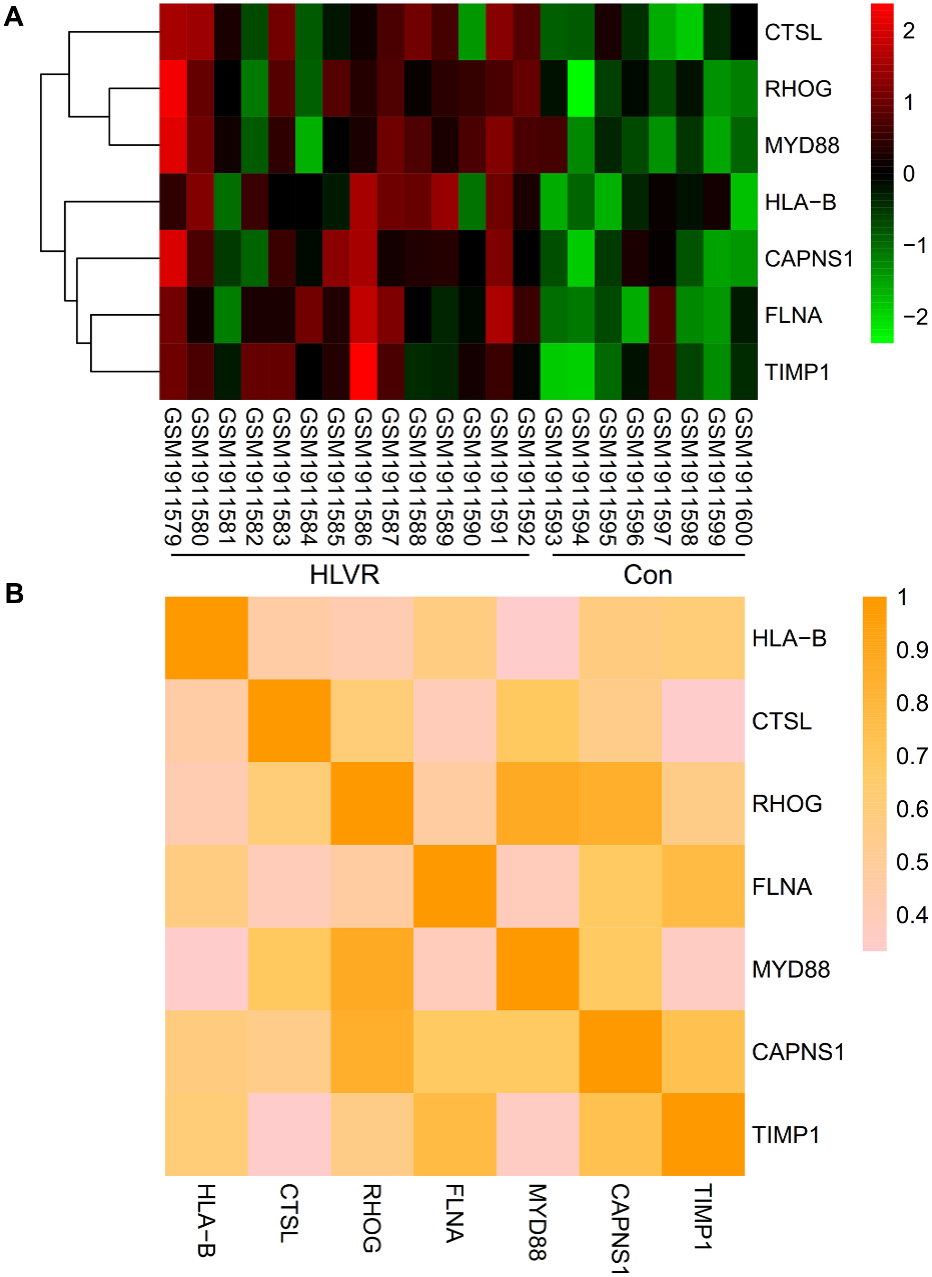
**Expression and correlation analysis of significant hub genes.** (**A**) A heatmap showing the expression level of significant hub genes in the GSE74144 dataset. (**B**) Correlations among all the significant hub genes.

A heatmap was generated to show the expression levels of significant hub genes in the GSE74144 dataset. When compared with the control white blood cell samples, the expression of HLA-B, CTSL, RHOG, FLNA, MYD88, CAPNS1, and TIMP1 were up-regulated in the HLVR white blood cell samples (Figure 5A). The correlations among all significant hub genes are shown in Figure 5B.

### Identification of hub genes

**Figure 6:**
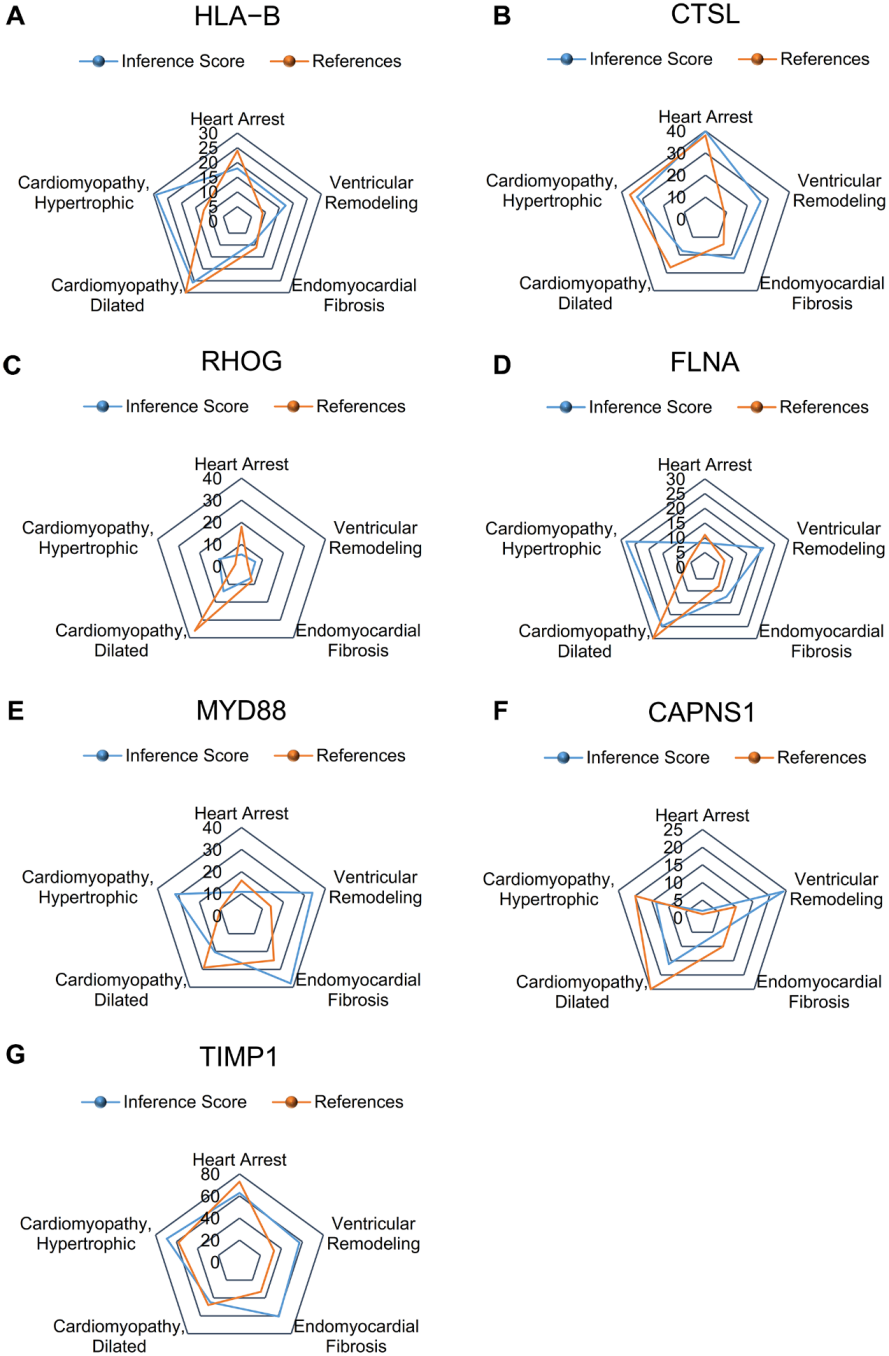
**Relationship to ventricular remodeling related to significant hub genes, based on the CTD.** (**A**) HLA-B, (**B**) CTSL, (**C**) RHOG, (**D**) FLNA, (**E**) MYD88, (**F**) CAPNS1, and (**G**) TIMP1.

The CTD showed that significant hub genes targeted ventricular remodeling; these data are shown in Figure 6A–6G.

### Differential expression analysis of HLA-B

The differential expression analysis result for HLA-B was log FC=-0.596, p=0.037 (Supplementary Figure 1A).

### WGCNA

In our study, WGCNA was conducted using the R package “WGCNA”. The selection of the soft-thresholding power was an important step in the WGCNA. Network topology analysis was performed to identify the soft-thresholding power, which was set to 17. A hierarchical clustering tree of all genes was constructed, and 12 important modules were generated (Supplementary Figure 1B). In addition, the dendrogram and heatmap of the genes showed that there were no significant differences in the interactions among different modules, demonstrating that there was a high degree of independence between these modules (Supplementary Figure 1C). HLA-B was in the brown module, and the correlation coefficient between HLA-B and ventricular remodeling was 0.67 (Supplementary Figure 1D). Interactions between HLA-B and other genes in the brown module were then analyzed (Supplementary Figure 1E). Module membership in the brown module is shown in Supplementary Figure 1F.

### Functional and pathway enrichment analysis of the brown module

The enrichment results of the GO analysis of the brown module performed by Metascape were mainly enriched in “ribonucleoprotein complex binding” and “catalytic activity, acting on RNA” (Supplementary Figure 1G). The enrichment results of the KEGG analysis were mainly enriched in “MAPK signaling pathway”, “phagosome”, “Fc gamma R-mediated phagocytosis”, and others (Supplementary Figure 1H). The enrichment heatmaps of the GO and KEGG analyses are shown in Supplementary Figure 1I.

### Functional and pathway enrichment analysis of HLA-B

GO enrichment in the HLA-B high-expression group was mainly enriched in “cardiolipin metabolic process” and “cell migration involved in heart development” (Supplementary Figure 2A). GO enrichment in the HLA-B low-expression group was mainly enriched in “cardiac epithelial to mesenchymal transition” and “cell migration involved in heart development” (Supplementary Figure 2B). The enrichment results of the KEGG analysis for the HLA-B high-expression group were mainly enriched in “MAPK signaling pathway”, “regulation of autophagy”, and others (Supplementary Figure 2C). The enrichment results of the KEGG analysis for the HLA-B low-expression group were enriched in “dilated cardiomyopathy” and “hypertrophic cardiomyopathy (HCM)” (Supplementary Figure 2D).

### Identification of hub genes

The ROC curve of HLA-B is shown in Supplementary Figure 2E. The CTD showed HLA-B was associated with ventricular remodeling, as shown in Supplementary Figure 2F.

### Prediction of ceRNA associated with HLA-B

The ceRNAs that regulate HLA-B were screened out using starBase (Supplementary Figure 2G).

### Statistical analysis and baseline clinical characteristics of the two groups

Based on univariate logistic proportional regression analysis, HLA-B had an effect on ventricular remodeling (P<0.05, Supplementary Table 2). At the same time, we analyzed the baseline clinical characteristics of the two groups. These results showed no deference in the baseline clinical characteristics of the two groups, which included sex, age, smoke, drinking, systolic blood pressure, diastolic blood pressure, mean arterial pressure, ejection fraction, left ventricular end-diastolic dimension (LVEDD), left Ventricular Posterior Wall Thickness (LVPWT), interventricular septum thickness (IVST); and the baseline medications including beta blockers, aldosterone receptor antagonist, angiotensin converting enzyme inhibitor (ACEI)/angiotensin II receptor blocker (ARB), Angiotensin receptor neprilysin inhibitor (ARNI) (P>0.05, Supplementary Table 3).

### Identification of candidate molecule drugs for HLA-B

Small molecule drugs for ventricular remodeling that could target HLA-B were screened using the CMap database. The small molecule drugs were filtered by the number of instances (n>3) and P-value <0.05, as shown in Supplementary Table 4.

### Results of the RT-qPCR analysis

**Figure 7:**
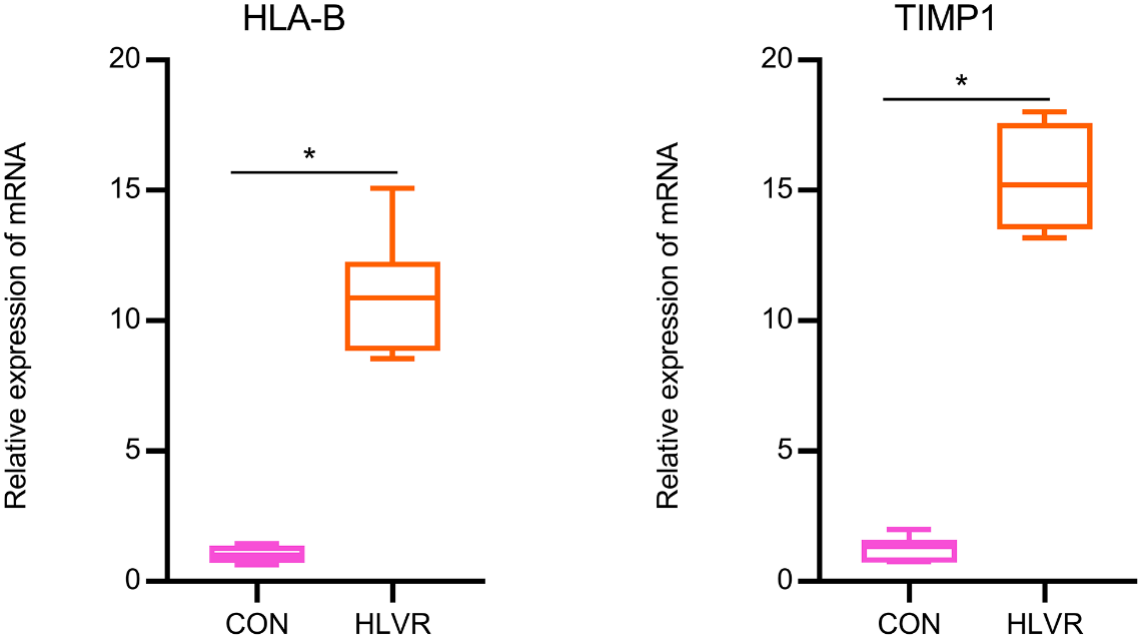
**The relative expression of HLA-B and TIMP1 by RT-qPCR analysis.** *P< 0.05.

According to the above expression analysis, HLA-B and TIMP1 were significantly up-regulated in HLVR samples. As shown in Figure 7, the relative expression levels of HLA-B and TIMP1 were significantly higher in HLVR samples compared with the control groups. This result demonstrated that HLA-B and TIMP1 might be considered as biomarkers for HLVR (Figure 7).

## DISCUSSION

In the present study, adopting a bioinformatics approach and confirming our findings on a small cohort of hypertensive patients, we found that HLA-B and TIMP1 genes are associated with hypertension-related ventricular remodeling.

Hypertension is the most important risk factor for cardiovascular and cerebrovascular diseases in the world today, threatening human health and causing many deaths [1]. Basic research into ventricular remodeling caused by hypertension is now based on the fundamentals of genomics. It has very important practical significance for the prevention and control of ventricular remodeling and may lead to significant breakthroughs [7]. Our team carried out in-depth microarray data mining and verification, aimed at the regulation mechanism of ventricular remodeling caused by hypertension.

Through the gene expression profile of the GSE74144 dataset, this research used bioinformatics analysis to screen DEGs and found 47 DEGs between control and HLVR samples. HLA-B, CTSL, RHOG, FLNA, MYD88, CAPNS1, and TIMP1 were identified as significant hub genes. When compared with the control white blood cell samples, the expression of HLA-B, CTSL, RHOG, FLNA, MYD88, CAPNS1, and TIMP1 were up-regulated in the HLVR white blood cell samples.

HLA (human leukocyte antigen) is the expression product of human major histocompatibility complex (MHC), a group of tightly linked genes that comprise the most complex polymorphic system known in the human body; they are located in the short arm of human chromosome 6 and encode the major histocompatibility antigen [12]. These genes are related to the immune response, inflammation, and transplant rejection. As a subtype of HLA, the main function of HLA-B is to recognize and present endogenous antigens, bind to the co-receptor CD8, and limit self-recognition reactions of cytotoxic T lymphocyte epitopes [12]. Johnson et al. found that HLA-B increased in patients with hypertension in the African population [13]. Wiktor et al. reported that HLA-B expression was not only elevated but also strongly correlated with the onset of hypertension in patients with hypertension in Poland [14]. Lenna et al. suggested that HLA-B could lead to disorders in the expression of inflammation reaction, oxide stress, and proliferation in patients with pulmonary arterial hypertension, thus proposing an underlying role for HLA-B in the occurrence and development of hypertension [15], which was similar to the findings of our study. HLA-B was significantly overexpressed in leukocytes of patients with hypertension-induced ventricular remodeling. During the process of long-term hypertension, the myocardial inflammation response is enhanced, which in turn activates the HLA immune system, resulting in increased HLA-B expression.

Tissue inhibitors of matrix metalloproteinases (TIMPs) maintain the dynamic balance of cardiac extracellular matrix (ECM) by inhibiting the activation of matrix metalloproteinases (MMPs). TIMP1 is one of the main subtypes of TIMPs. The primary function of MMPs is to degrade ECM. When TIMP expression is elevated, the activation of MMPs is over-inhibited, and there is degradation of the biological effects of ECM, which can lead to the over-deposition of ECM and lead to myocardial fibrosis. Lopez et al. demonstrated that plasma TIMP1 levels in patients with hypertension are closely related to the degree of myocardial fibrosis in endocardial biopsies [16]. Lindsay et al. showed that TIMP1 is a biomarker for myocardial fibrosis in patients with hypertension [17]. In addition to clinical studies showing that TIMP1 is involved in the occurrence and development of myocardial fibrosis, there have also been animal experiments showing the molecular mechanism of how elevated TIMP1 promotes hypertensive myocardial fibrosis. For example, Takawale et al. used *in vitro* analysis to evaluate the role of TIMP1 in myocardial fibrosis by using TIMP1 gene knockout mice, animal models of myocardial fibrosis caused by hypertension, and myocardial fibrosis in patients with dilated cardiomyopathy (DCM). TIMP1 knockout significantly decreased myocardial fibrosis. Simultaneously, TIMP1 might be a new biomarker for fibrosis, as it is a newly discovered mechanism. In addition to inhibiting the activation of matrix metalloproteinases, TIMP1 could also play an important role in myocardial fibroblasts. Through CD63 (TIMP1 cell-surface receptors), TIMP1 and integrin beta 1 could promote activation and nuclear transfer of Smad2/3 and the beta serial protein, resulting in the deposition of types I and III collagen and subsequently myocardial fibrosis [18]. A study by Ghosh et al. demonstrated that the increased expression of TIMP1 is involved in the process of endothelial-to-mesenchymal transition (ENDMT) and cardiac fibrosis [19]. Therefore, the above studies all support the results obtained from the bioinformatics analyses described here. The expression of TIMP1 is up-regulated in patients with hypertension-related myocardial fibrosis and ventricular remodeling.

In conclusion, our study evaluated the ability of HLA-B and TIMP1 to participate in ventricular remodeling caused by hypertension. More explorations of a similar kind are advocated for the future.

### Limitations

Although the team conducted in-depth mining and analysis of public data, this study also had some shortcomings. Due to the limited datasets available for ventricular remodeling, the sample size included in this paper was small. Although the correlation between hub genes and ventricular remodeling was statistically verified and patient samples were used in this study, animal experiments were not performed. However, due to the difficulty of sample collection and insufficient funds, we were unable to complete these in a short period of time especially in this current pandemic. In the described population, male sex is highly prevalent in the ventricular remodeling group (6:1 male:female with remodeling vs 4:1). The cross-sectional setting of the RT-PCR observation is another one limitation.

### Future directions

In future research, the sample size must be increased to obtain more accurate results. Comprehensive verification using animal experiments will also be needed to better understand the molecular mechanisms of ventricular remodeling caused by hypertension.

## CONCLUSIONS

In summary, bioinformatics is a valuable method for exploring the molecular mechanism of the occurrence and development of HLVR. Furthermore, our analyses identified DEGs between control and HLVR samples, which might participate in the mechanism of HLVR. In particular, the HLA-B and TIMP1 genes could provide a novel research target for the diagnosis and treatment of HLVR. HLA-B and TIMP1 have potential clinical applications and may play an important role in future studies.

## Supplementary Material

Supplementary Figures

Supplementary Tables
