# Supplementary material for: HLA-B and TIMP1 as hub genes of the ventricular remodeling caused by hypertension
Source: Aging (Albany NY). 2024 May 9;16(9):8260–78. doi: 10.18632/aging.205816 (PMC11132017; doi:10.18632/aging.205816)
Supplement: Supplementary Figures [file aging-16-205816-s001.pdf]

SUPPLEMENTARY FIGURES

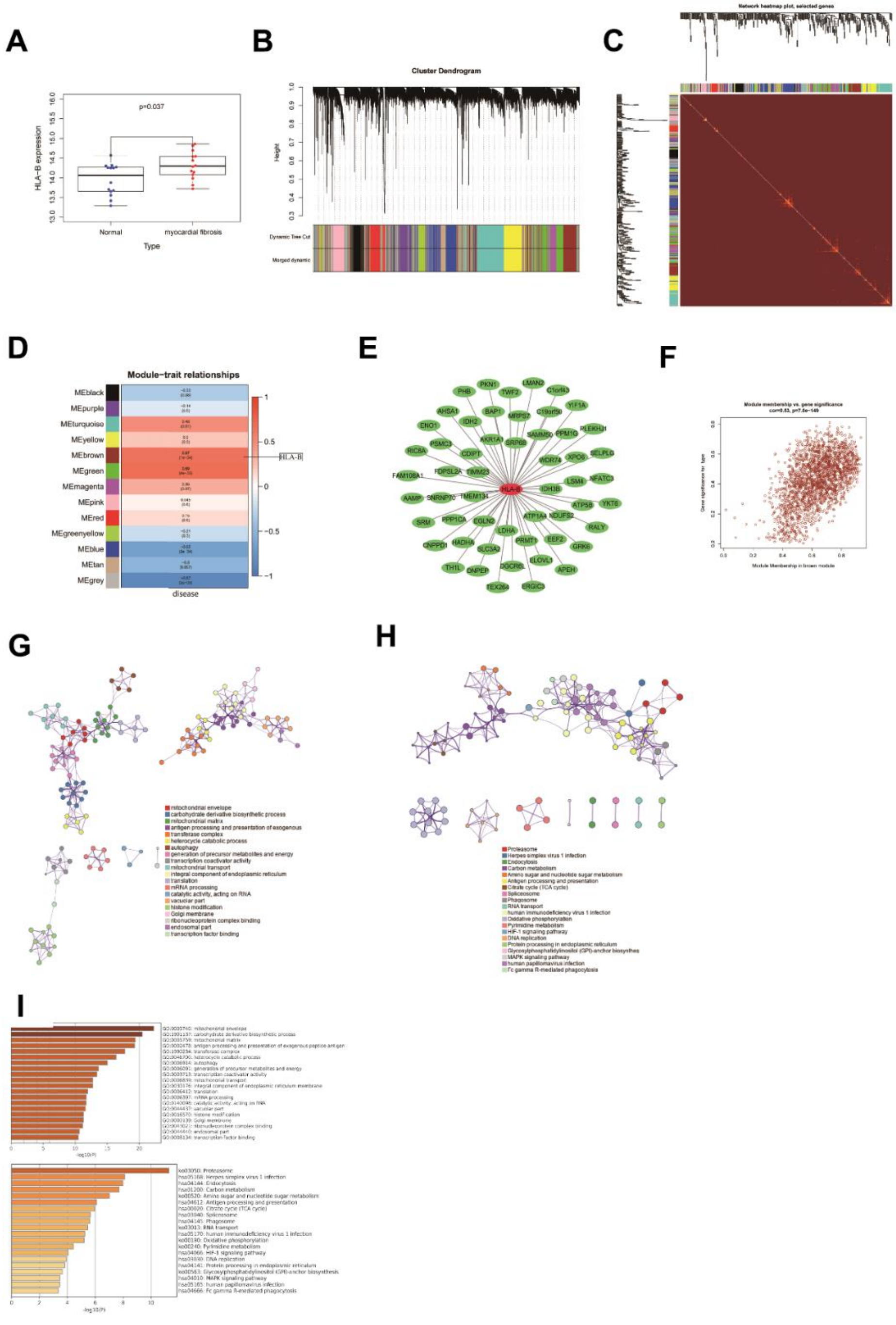

Supplementary Figure 1. Differential expression analysis and WGCNA of the genes in the GSE74144 dataset. (**A**) A box diagram of HLA-B showing differential expression between the ventricular remodeling and control groups. (**B**) A repeated hierarchical clustering tree of all genes; HLA-B is in the brown module. (**C**) A dendrogram and heatmap showing all the genes. (**D**) The associations between clinical traits and the modules and the correlation between the brown module and ventricular remodeling is 0.67. (**E**) Interrelationships between HLA-B and the genes in the brown module. (**F**) Module membership in the brown module. (**G**) GO enrichment analysis of the brown module genes using Metascape. (**H**) KEGG analysis. (**I**) A heatmap showing GO and KEGG analyses using Metascape.

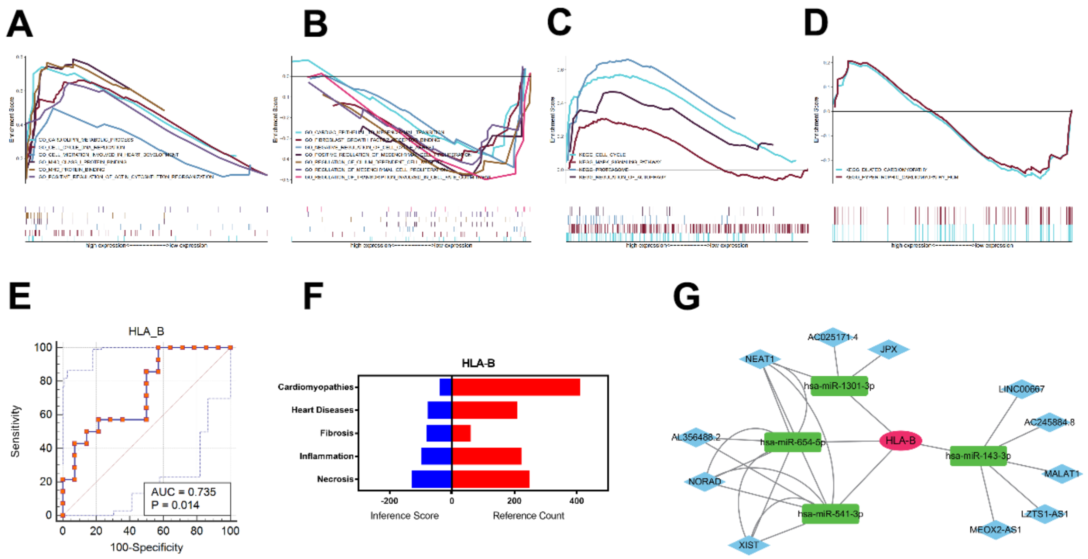

Supplementary Figure 2. Gene functional enrichment analysis of HLA-B by GSEA. (**A**, **B**) GO analyses by GSEA. (**C**, **D**) KEGG analyses by GSEA. (**E**) ROC curves of HLA-B. (**F**) Relationships between the ventricular remodeling group and the control group related to HLA-B, based on the CTD. (**G**) Predicting the ceRNA network mechanism of HLA-B using starBase.
