# Supplementary material for: HLA-B and TIMP1 as hub genes of the ventricular remodeling caused by hypertension
Source: Aging (Albany NY). 2024 May 9;16(9):8260–78. doi: 10.18632/aging.205816 (PMC11132017; doi:10.18632/aging.205816)
Supplement: Supplementary Tables [file aging-16-205816-s002.pdf]

SUPPLEMENTARY TABLES

Supplementary Table 1. Primers and their sequences for RT-PCR analysis.

Primer	Sequence (5′–3′)
HLA-B-hF	GAACACACAGATCTACAAGGCCC
HLA-B-hR	CGTAGGCGTACTGGTCATGC
TIMP1-hF	CAATTCCGACCTCGTCATCAG
TIMP1-hR	GGTTGTGGGACCTGTGGAAGTA

HLA, Human leukocyte antigen; TIMPs, Tissue inhibitor of matrix metalloproteinases.

Supplementary Table 2. The genes and their effect on ventricular remodeling based on univariate logistic proportional regression analysis.

GENE	OR	95% CI	P
HLA-B	12.996	1.234 - 136.895	0.033

HLA, Human leukocyte antigen; OR, odds ratio; 95% CI, 95% confidence interval.

Supplementary Table 3. Based information of participants with hypertension.

Characteristics		Ventricular remodeling		P
		No (%)	Yes (%)	
Sex				0.237
Male	10	4 (28.6%)	6 (42.9%)	
Female	4	3 (21.4%)	1 (7.1%)	
Age (years)	14	66.571±15.361	70.857±6.122	0.506
Smoke				
No	9	5 (35.7%)	4 (28.6%)	0.577
Yes	5	2 (14.3%)	3 (21.4%)	
Drinking				
No	10	5 (35.7%)	5 (35.7%)	1.000
Yes	4	2 (14.3%)	2 (14.3%)	
Treatment with beta blockers				
No	3	2 (14.3%)	1 (7.1%)	0.515
Yes	11	5 (35.7%)	6 (42.9%)	
Treatment with aldosterone receptor antagonist (MRA)				
No	6	3 (21.4%)	3 (21.4%)	1.000
Yes	8	4 (28.6%)	4 (28.6%)	
Treatment with ACEI/ARB				
No	10	6 (42.9%)	4 (28.6%)	0.237
Yes	4	1 (7.1%)	3 (21.4%)	
Treatment with **ARNI**				
No	10	5 (35.7%)	5 (35.7%)	1.000
Yes	4	2 (14.3%)	2 (14.3%)	
Whether have hypertension				
No	0	0 (0.0%)	0 (0.0%)	1.000
Yes	14	7 (50%)	7 (50%)	
Systolic pressure (mmHg)	14	138.714±22.962	141.143±14.634	0.817
Diastolic pressure (mmHg)	14	84.429±7.786	79.429±7.721	0.251
Mean arterial pressure (mmHg)	14	102.52±10.65	100.00±7.53	0.539
Ejection fraction (%)	14	51.57±11.31	52.86±10.68	0.973
LVEDD (mm)	14	53.86±8.82	53.29±6.21	0.398
LVPWT (mm)	14	10.57±1.81	10.29±0.76	0.083
IVST (mm)	14	11.71±2.93	10.43±1.90	0.576

Fisher's Exact Test was used for categorical variable, independent-samples T test (student's t-test) for continuous variable. P-values refer to the significance between ventricular remodeling and different characteristics. LVEDD: left ventricular end-diastolic dimension; LVPWT: Left Ventricular Posterior Wall Thickness; IVST: interventricular septum thickness.

Supplementary Table 4. A summary of connectivity map that may regulate HLA-B.

	cmap name	mean	n	enrichment	p
1	isoflupredone	-0.818	3	-0.945	0.00028
2	Gly-His-Lys	-0.8	3	-0.921	0.00084
3	ciclacillin	0.806	4	0.829	0.00131
4	thiamphenicol	-0.73	5	-0.748	0.00188
5	ethisterone	-0.496	6	-0.688	0.0022
6	adiphenine	-0.44	5	-0.704	0.00501
7	verteporfin	0.778	3	0.861	0.00503
8	amantadine	-0.734	4	-0.775	0.00527
9	acepromazine	0.746	4	0.769	0.00553
10	timolol	-0.751	4	-0.771	0.00557

HLA, Human leukocyte antigen.
